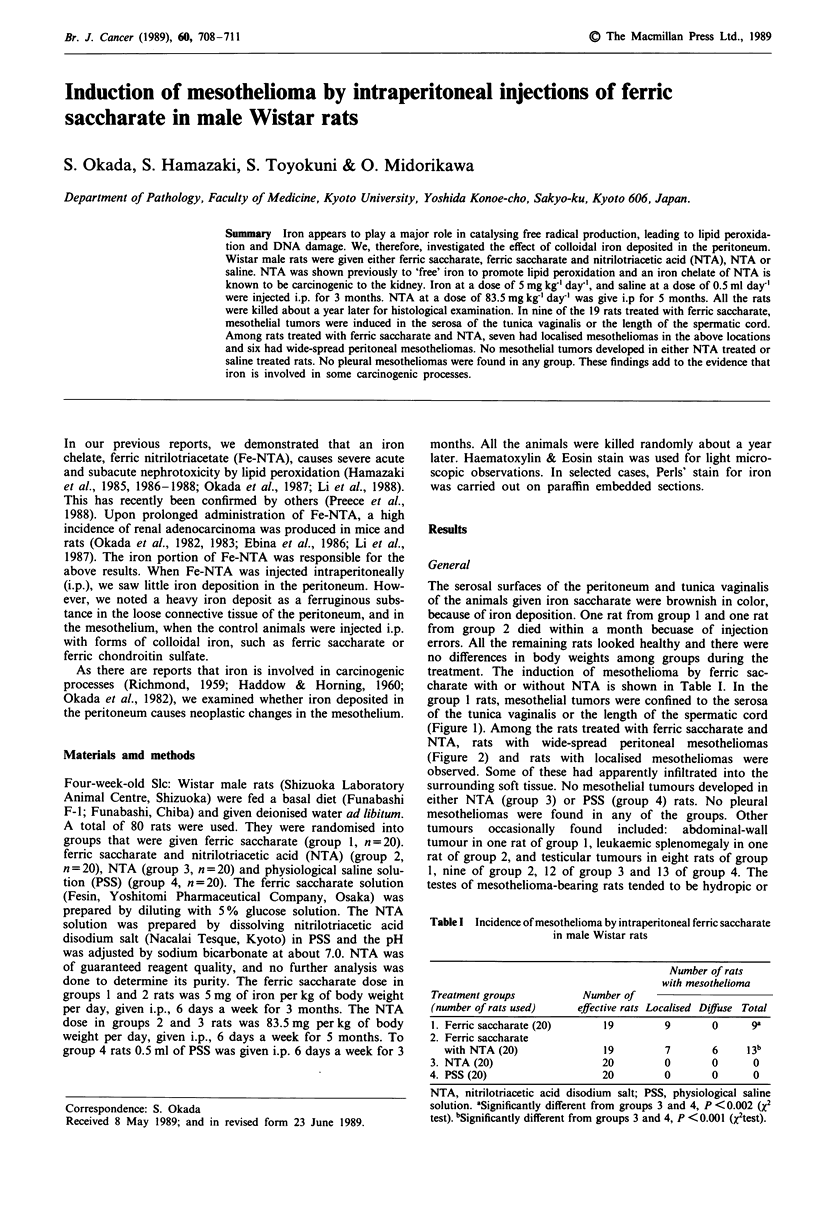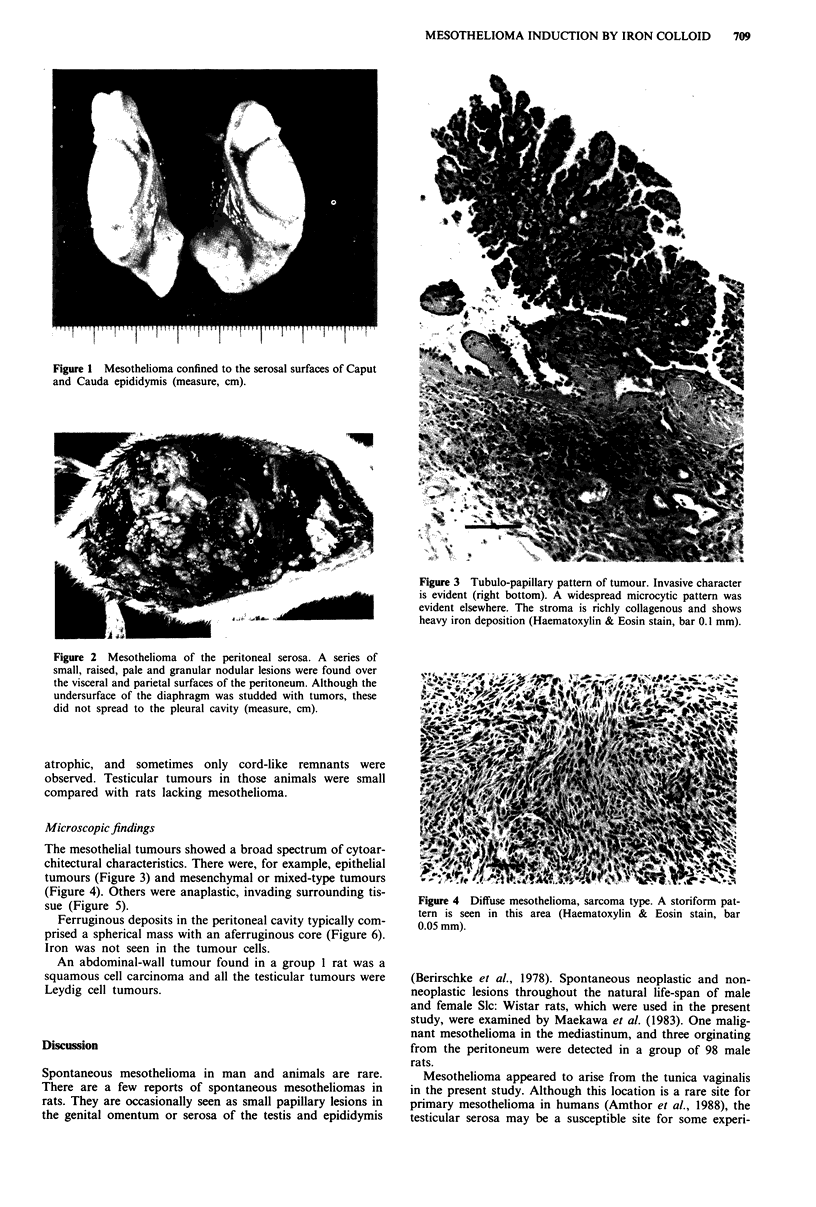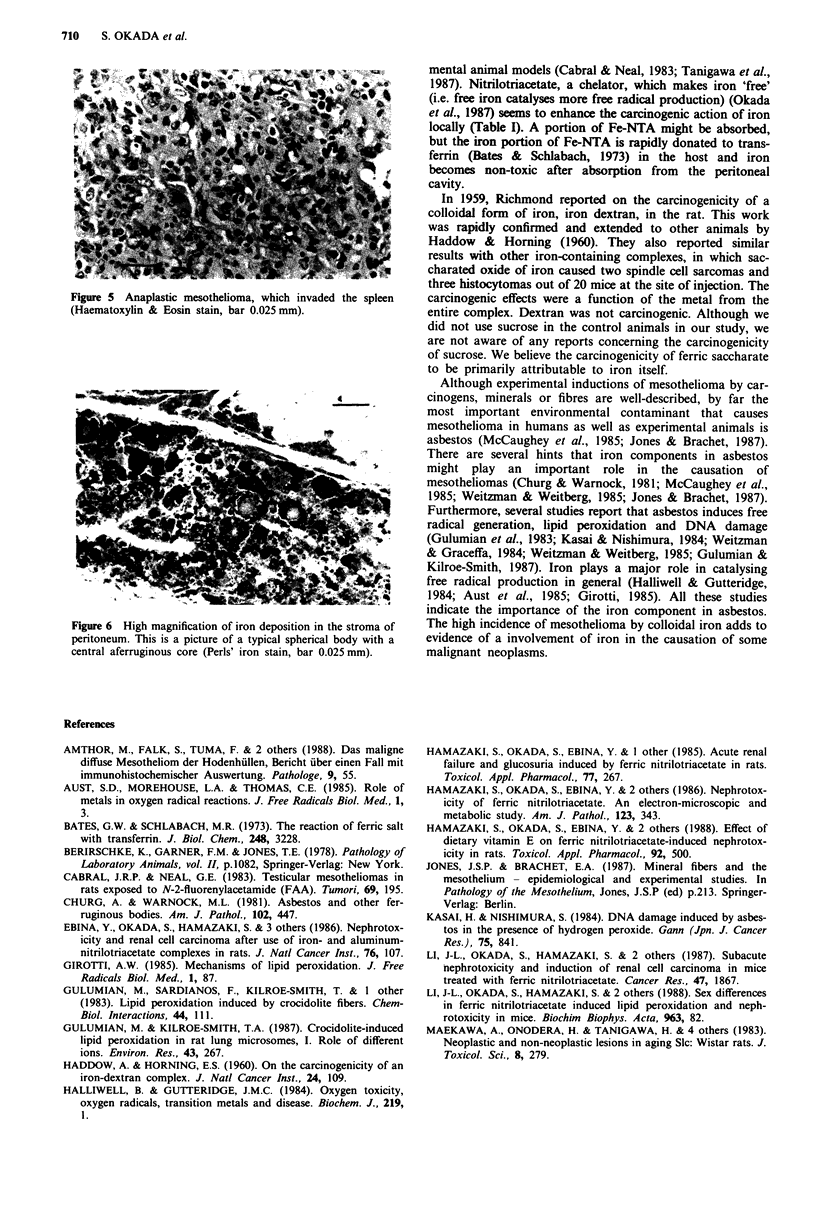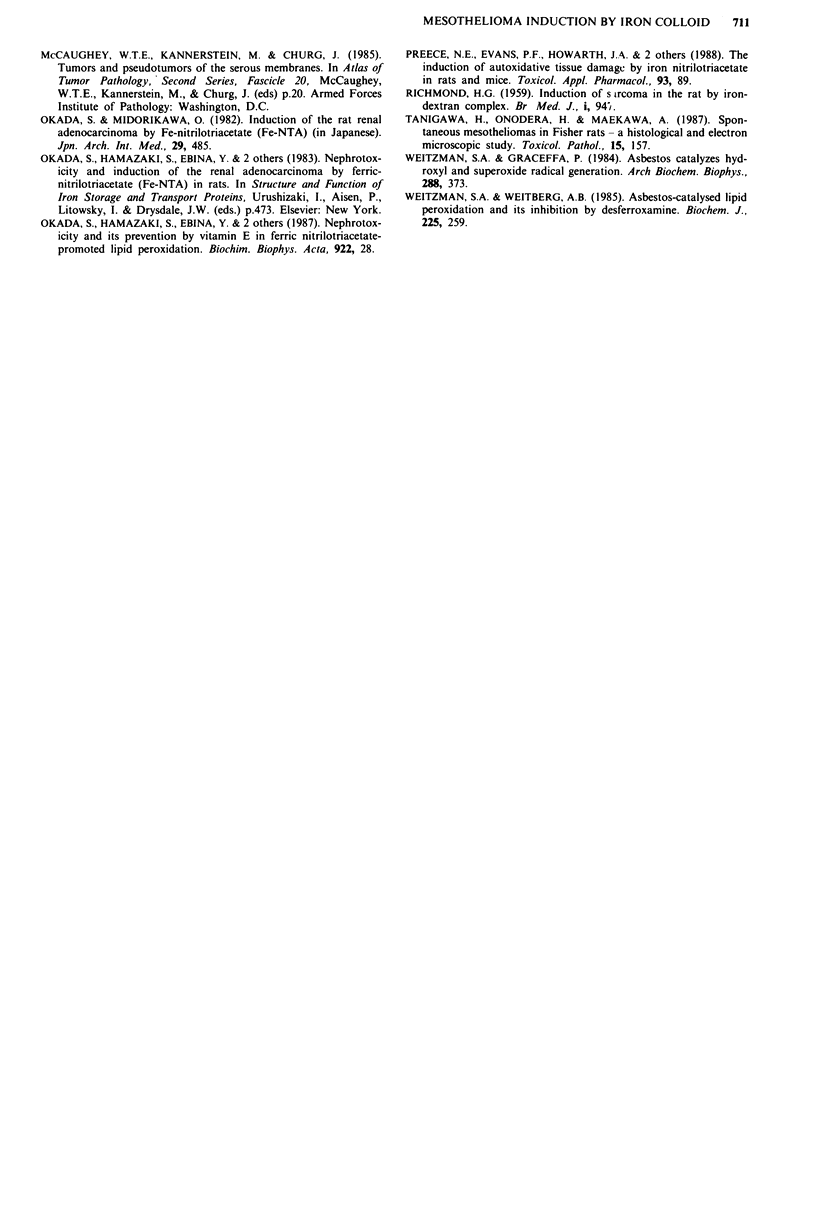# Induction of mesothelioma by intraperitoneal injections of ferric saccharate in male Wistar rats.

**DOI:** 10.1038/bjc.1989.344

**Published:** 1989-11

**Authors:** S. Okada, S. Hamazaki, S. Toyokuni, O. Midorikawa

**Affiliations:** Department of Pathology, Faculty of Medicine, Kyoto University, Japan.

## Abstract

**Images:**


*Br. J. Cancer* (1989), **60**, 708-711 © The Macmillan Press Ltd., 1989

### Induction of mesothelioma by intraperitoneal injections of ferric saccharate in male Wistar rats

S. Okada, S. Hamazaki, S. Toyokuni & O. Midorikawa

*Department of Pathology, Faculty of Medicine, Kyoto University, Yoshida Konoe-cho, Sakyo-ku, Kyoto 606, Japan.*

**Summary** Iron appears to play a major role in catalysing free radical production, leading to lipid peroxidation and DNA damage. We, therefore, investigated the effect of colloidal iron deposited in the peritoneum. Wistar male rats were given either ferric saccharate, ferric saccharate and nitrilotriacetic acid (NTA), NTA or saline. NTA was shown previously to 'free' iron to promote lipid peroxidation and an iron chelate of NTA is known to be carcinogenic to the kidney. Iron at a dose of 5 mg $kg^{-1}$ $day^{-1}$, and saline at a dose of 0.5 ml $day^{-1}$ were injected i.p. for 3 months. NTA at a dose of 83.5 mg $kg^{-1}$ $day^{-1}$ was give i.p for 5 months. All the rats were killed about a year later for histological examination. In nine of the 19 rats treated with ferric saccharate, mesothelial tumors were induced in the serosa of the tunica vaginalis or the length of the spermatic cord. Among rats treated with ferric saccharate and NTA, seven had localised mesotheliomas in the above locations and six had wide-spread peritoneal mesotheliomas. No mesothelial tumors developed in either NTA treated or saline treated rats. No pleural mesotheliomas were found in any group. These findings add to the evidence that iron is involved in some carcinogenic processes.

In our previous reports, we demonstrated that an iron chelate, ferric nitrilotriacetate (Fe-NTA), causes severe acute and subacute nephrotoxicity by lipid peroxidation (Hamazaki *et al.*, 1985, 1986-1988; Okada *et al.*, 1987; Li *et al.*, 1988). This has recently been confirmed by others (Preece *et al.*, 1988). Upon prolonged administration of Fe-NTA, a high incidence of renal adenocarcinoma was produced in mice and rats (Okada *et al.*, 1982, 1983; Ebina *et al.*, 1986; Li *et al.*, 1987). The iron portion of Fe-NTA was responsible for the above results. When Fe-NTA was injected intraperitoneally (i.p.), we saw little iron deposition in the peritoneum. However, we noted a heavy iron deposit as a ferruginous substance in the loose connective tissue of the peritoneum, and in the mesothelium, when the control animals were injected i.p. with forms of colloidal iron, such as ferric saccharate or ferric chondroitin sulfate.

As there are reports that iron is involved in carcinogenic processes (Richmond, 1959; Haddow & Horning, 1960; Okada *et al.*, 1982), we examined whether iron deposited in the peritoneum causes neoplastic changes in the mesothelium.

### Materials amd methods

Four-week-old Slc: Wistar male rats (Shizuoka Laboratory Animal Centre, Shizuoka) were fed a basal diet (Funabashi F-1; Funabashi, Chiba) and given deionised water *ad libitum*. A total of 80 rats were used. They were randomised into groups that were given ferric saccharate (group 1, $n=20$). ferric saccharate and nitrilotriacetic acid (NTA) (group 2, $n=20$), NTA (group 3, $n=20$) and physiological saline solution (PSS) (group 4, $n=20$). The ferric saccharate solution (Fesin, Yoshitomi Pharmaceutical Company, Osaka) was prepared by diluting with 5% glucose solution. The NTA solution was prepared by dissolving nitrilotriacetic acid disodium salt (Nacalai Tesque, Kyoto) in PSS and the pH was adjusted by sodium bicarbonate at about 7.0. NTA was of guaranteed reagent quality, and no further analysis was done to determine its purity. The ferric saccharate dose in groups 1 and 2 rats was 5 mg of iron per kg of body weight per day, given i.p., 6 days a week for 3 months. The NTA dose in groups 2 and 3 rats was 83.5 mg per kg of body weight per day, given i.p., 6 days a week for 5 months. To group 4 rats 0.5 ml of PSS was given i.p. 6 days a week for 3 months. All the animals were killed randomly about a year later. Haematoxylin & Eosin stain was used for light microscopic observations. In selected cases, Perls' stain for iron was carried out on paraffin embedded sections.

Correspondence: S. Okada
Received 8 May 1989; and in revised form 23 June 1989.

### Results

### *General*

The serosal surfaces of the peritoneum and tunica vaginalis of the animals given iron saccharate were brownish in color, because of iron deposition. One rat from group 1 and one rat from group 2 died within a month becuase of injection errors. All the remaining rats looked healthy and there were no differences in body weights among groups during the treatment. The induction of mesothelioma by ferric saccharate with or without NTA is shown in Table I. In the group 1 rats, mesothelial tumors were confined to the serosa of the tunica vaginalis or the length of the spermatic cord (Figure 1). Among the rats treated with ferric saccharate and NTA, rats with wide-spread peritoneal mesotheliomas (Figure 2) and rats with localised mesotheliomas were observed. Some of these had apparently infiltrated into the surrounding soft tissue. No mesothelial tumours developed in either NTA (group 3) or PSS (group 4) rats. No pleural mesotheliomas were found in any of the groups. Other tumours occasionally found included: abdominal-wall tumour in one rat of group 1, leukaemic splenomegaly in one rat of group 2, and testicular tumours in eight rats of group 1, nine of group 2, 12 of group 3 and 13 of group 4. The testes of mesothelioma-bearing rats tended to be hydropic or

**Table I** Incidence of mesothelioma by intraperitoneal ferric saccharate in male Wistar rats

| *Treatment groups (number of rats used)* | *Number of effective rats* | *Number of rats with mesothelioma* | | |
|---|---|---|---|---|
| | | *Localised* | *Diffuse* | *Total* |
| 1. Ferric saccharate (20) | 19 | 9 | 0 | 9[a] |
| 2. Ferric saccharate with NTA (20) | 19 | 7 | 6 | 13[b] |
| 3. NTA (20) | 20 | 0 | 0 | 0 |
| 4. PSS (20) | 20 | 0 | 0 | 0 |

NTA, nitrilotriacetic acid disodium salt; PSS, physiological saline solution. [a]Significantly different from groups 3 and 4, $P<0.002$ ($\chi^2$ test). [b]Significantly different from groups 3 and 4, $P<0.001$ ($\chi^2$test).

**Figure 1** Mesothelioma confined to the serosal surfaces of Caput and Cauda epididymis (measure, cm).

**Figure 2** Mesothelioma of the peritoneal serosa. A series of small, raised, pale and granular nodular lesions were found over the visceral and parietal surfaces of the peritoneum. Although the undersurface of the diaphragm was studded with tumors, these did not spread to the pleural cavity (measure, cm).

atrophic, and sometimes only cord-like remnants were observed. Testicular tumours in those animals were small compared with rats lacking mesothelioma.

### *Microscopic findings*

The mesothelial tumours showed a broad spectrum of cytoarchitectural characteristics. There were, for example, epithelial tumours (Figure 3) and mesenchymal or mixed-type tumours (Figure 4). Others were anaplastic, invading surrounding tissue (Figure 5).

Ferruginous deposits in the peritoneal cavity typically comprised a spherical mass with an aferruginous core (Figure 6). Iron was not seen in the tumour cells.

An abdominal-wall tumour found in a group 1 rat was a squamous cell carcinoma and all the testicular tumours were Leydig cell tumours.

### Discussion

Spontaneous mesothelioma in man and animals are rare. There are a few reports of spontaneous mesotheliomas in rats. They are occasionally seen as small papillary lesions in the genital omentum or serosa of the testis and epididymis

**Figure 3** Tubulo-papillary pattern of tumour. Invasive character is evident (right bottom). A widespread microcytic pattern was evident elsewhere. The stroma is richly collagenous and shows heavy iron deposition (Haematoxylin & Eosin stain, bar 0.1 mm).

**Figure 4** Diffuse mesothelioma, sarcoma type. A storiform pattern is seen in this area (Haematoxylin & Eosin stain, bar 0.05 mm).

(Berirschke *et al.*, 1978). Spontaneous neoplastic and non-neoplastic lesions throughout the natural life-span of male and female Slc: Wistar rats, which were used in the present study, were examined by Maekawa *et al.* (1983). One malignant mesothelioma in the mediastinum, and three orginating from the peritoneum were detected in a group of 98 male rats.

Mesothelioma appeared to arise from the tunica vaginalis in the present study. Although this location is a rare site for primary mesothelioma in humans (Amthor *et al.*, 1988), the testicular serosa may be a susceptible site for some experi-

**Figure 5** Anaplastic mesothelioma, which invaded the spleen (Haematoxylin & Eosin stain, bar 0.025 mm).

**Figure 6** High magnification of iron deposition in the stroma of peritoneum. This is a picture of a typical spherical body with a central aferruginous core (Perls' iron stain, bar 0.025 mm).

mental animal models (Cabral & Neal, 1983; Tanigawa et al., 1987). Nitrilotriacetate, a chelator, which makes iron 'free' (i.e. free iron catalyses more free radical production) (Okada et al., 1987) seems to enhance the carcinogenic action of iron locally (Table I). A portion of Fe-NTA might be absorbed, but the iron portion of Fe-NTA is rapidly donated to transferrin (Bates & Schlabach, 1973) in the host and iron becomes non-toxic after absorption from the peritoneal cavity.

In 1959, Richmond reported on the carcinogenicity of a colloidal form of iron, iron dextran, in the rat. This work was rapidly confirmed and extended to other animals by Haddow & Horning (1960). They also reported similar results with other iron-containing complexes, in which saccharated oxide of iron caused two spindle cell sarcomas and three histocytomas out of 20 mice at the site of injection. The carcinogenic effects were a function of the metal from the entire complex. Dextran was not carcinogenic. Although we did not use sucrose in the control animals in our study, we are not aware of any reports concerning the carcinogenicity of sucrose. We believe the carcinogenicity of ferric saccharate to be primarily attributable to iron itself.

Although experimental inductions of mesothelioma by carcinogens, minerals or fibres are well-described, by far the most important environmental contaminant that causes mesothelioma in humans as well as experimental animals is asbestos (McCaughey et al., 1985; Jones & Brachet, 1987). There are several hints that iron components in asbestos might play an important role in the causation of mesotheliomas (Churg & Warnock, 1981; McCaughey et al., 1985; Weitzman & Weitberg, 1985; Jones & Brachet, 1987). Furthermore, several studies report that asbestos induces free radical generation, lipid peroxidation and DNA damage (Gulumian et al., 1983; Kasai & Nishimura, 1984; Weitzman & Graceffa, 1984; Weitzman & Weitberg, 1985; Gulumian & Kilroe-Smith, 1987). Iron plays a major role in catalysing free radical production in general (Halliwell & Gutteridge, 1984; Aust et al., 1985; Girotti, 1985). All these studies indicate the importance of the iron component in asbestos. The high incidence of mesothelioma by colloidal iron adds to evidence of a involvement of iron in the causation of some malignant neoplasms.

### References

AMTHOR, M., FALK, S., TUMA, F. & 2 others (1988). Das maligne diffuse Mesotheliom der Hodenhüllen, Bericht über einen Fall mit immunohistochemischer Auswertung. *Pathologe*, **9**, 55.

AUST, S.D., MOREHOUSE, L.A. & THOMAS, C.E. (1985). Role of metals in oxygen radical reactions. *J. Free Radicals Biol. Med.*, **1**, 3.

BATES, G.W. & SCHLABACH, M.R. (1973). The reaction of ferric salt with transferrin. *J. Biol. Chem.*, **248**, 3228.

BERIRSCHKE, K., GARNER, F.M. & JONES, T.E. (1978). *Pathology of Laboratory Animals, vol. II*, p.1082, Springer-Verlag: New York.

CABRAL, J.R.P. & NEAL, G.E. (1983). Testicular mesotheliomas in rats exposed to *N*-2-fluorenylacetamide (FAA). *Tumori*, **69**, 195.

CHURG, A. & WARNOCK, M.L. (1981). Asbestos and other ferruginous bodies. *Am. J. Pathol.*, **102**, 447.

EBINA, Y., OKADA, S., HAMAZAKI, S. & 3 others (1986). Nephrotoxicity and renal cell carcinoma after use of iron- and aluminum-nitrilotriacetate complexes in rats. *J. Natl Cancer Inst.*, **76**, 107.

GIROTTI, A.W. (1985). Mechanisms of lipid peroxidation. *J. Free Radicals Biol. Med.*, **1**, 87.

GULUMIAN, M., SARDIANOS, F., KILROE-SMITH, T. & 1 other (1983). Lipid peroxidation induced by crocidolite fibers. *Chem-Biol. Interactions*, **44**, 111.

GULUMIAN, M. & KILROE-SMITH, T.A. (1987). Crocidolite-induced lipid peroxidation in rat lung microsomes, I. Role of different ions. *Environ. Res.*, **43**, 267.

HADDOW, A. & HORNING, E.S. (1960). On the carcinogenicity of an iron-dextran complex. *J. Natl Cancer Inst.*, **24**, 109.

HALLIWELL, B. & GUTTERIDGE, J.M.C. (1984). Oxygen toxicity, oxygen radicals, transition metals and disease. *Biochem. J.*, **219**, 1.

HAMAZAKI, S., OKADA, S., EBINA, Y. & 1 other (1985). Acute renal failure and glucosuria induced by ferric nitrilotriacetate in rats. *Toxicol. Appl. Pharmacol.*, **77**, 267.

HAMAZAKI, S., OKADA, S., EBINA, Y. & 2 others (1986). Nephrotoxicity of ferric nitrilotriacetate. An electron-microscopic and metabolic study. *Am. J. Pathol.*, **123**, 343.

HAMAZAKI, S., OKADA, S., EBINA, Y. & 2 others (1988). Effect of dietary vitamin E on ferric nitrilotriacetate-induced nephrotoxicity in rats. *Toxicol. Appl. Pharmacol.*, **92**, 500.

JONES, J.S.P. & BRACHET, E.A. (1987). Mineral fibers and the mesothelium – epidemiological and experimental studies. In *Pathology of the Mesothelium*, Jones, J.S.P (ed) p.213. Springer-Verlag: Berlin.

KASAI, H. & NISHIMURA, S. (1984). DNA damage induced by asbestos in the presence of hydrogen peroxide. *Gann (Jpn. J. Cancer Res.)*, **75**, 841.

LI, J-L., OKADA, S., HAMAZAKI, S. & 2 others (1987). Subacute nephrotoxicity and induction of renal cell carcinoma in mice treated with ferric nitrilotriacetate. *Cancer Res.*, **47**, 1867.

LI, J-L., OKADA, S., HAMAZAKI, S. & 2 others (1988). Sex differences in ferric nitrilotriacetate induced lipid peroxidation and nephrotoxicity in mice. *Biochim Biophys. Acta*, **963**, 82.

MAEKAWA, A., ONODERA, H. & TANIGAWA, H. & 4 others (1983). Neoplastic and non-neoplastic lesions in aging Slc: Wistar rats. *J. Toxicol. Sci.*, **8**, 279.

McCAUGHEY, W.T.E., KANNERSTEIN, M. & CHURG, J. (1985). Tumors and pseudotumors of the serous membranes. In *Atlas of Tumor Pathology, Second Series, Fascicle 20,* McCaughey, W.T.E., Kannerstein, M., & Churg, J. (eds) p.20. Armed Forces Institute of Pathology: Washington, D.C.

OKADA, S. & MIDORIKAWA, O. (1982). Induction of the rat renal adenocarcinoma by Fe-nitrilotriacetate (Fe-NTA) (in Japanese). *Jpn. Arch. Int. Med.,* **29,** 485.

OKADA, S., HAMAZAKI, S., EBINA, Y. & 2 others (1983). Nephrotoxicity and induction of the renal adenocarcinoma by ferric-nitrilotriacetate (Fe-NTA) in rats. In *Structure and Function of Iron Storage and Transport Proteins,* Urushizaki, I., Aisen, P., Litowsky, I. & Drysdale, J.W. (eds.) p.473. Elsevier: New York.

OKADA, S., HAMAZAKI, S., EBINA, Y. & 2 others (1987). Nephrotoxicity and its prevention by vitamin E in ferric nitrilotriacetate-promoted lipid peroxidation. *Biochim. Biophys. Acta,* **922,** 28.

PREECE, N.E., EVANS, P.F., HOWARTH, J.A. & 2 others (1988). The induction of autoxidative tissue damage by iron nitrilotriacetate in rats and mice. *Toxicol. Appl. Pharmacol.,* **93,** 89.

RICHMOND, H.G. (1959). Induction of sarcoma in the rat by iron-dextran complex. *Br Med. J.,* **i,** 947.

TANIGAWA, H., ONODERA, H. & MAEKAWA, A. (1987). Spontaneous mesotheliomas in Fisher rats – a histological and electron microscopic study. *Toxicol. Pathol.,* **15,** 157.

WEITZMAN, S.A. & GRACEFFA, P. (1984). Asbestos catalyzes hydroxyl and superoxide radical generation. *Arch Biochem. Biophys.,* **288,** 373.

WEITZMAN, S.A. & WEITBERG, A.B. (1985). Asbestos-catalysed lipid peroxidation and its inhibition by desferroxamine. *Biochem. J.,* **225,** 259.